# The Federal Menu Labeling Law and Twitter Discussions about Calories in the United States: An Interrupted Time-Series Analysis

**DOI:** 10.3390/ijerph182010794

**Published:** 2021-10-14

**Authors:** Yulin Hswen, Alyssa J. Moran, Siona Prasad, Anna Li, Denise Simon, Lauren Cleveland, Jared B. Hawkins, John S. Brownstein, Jason Block

**Affiliations:** 1 Department of Epidemiology and Biostatistics, University of California San Francisco, San Francisco, CA 94158, USA; 2 Bakar Computational Health Sciences Institute, University of California San Francisco, San Francisco, CA 94158, USA; 3 Computational Epidemiology Lab, Harvard Medical School, Boston, MA 02215, USA; Jared.Hawkins@childrens.harvard.edu (J.B.H.); John.Brownstein@childrens.harvard.edu (J.S.B.); 4 Innovation Program, Boston Children’s Hospital, Boston, MA 02215, USA; 5 Department of Health Policy and Management, Bloomberg School of Public Health, Johns Hopkins University, Baltimore, MA 21205, USA; amoran10@jhu.edu; 6 Harvard University, Cambridge, MA 02138, USA; sionaprasad@college.harvard.edu (S.P.); annali2@college.harvard.edu (A.L.); 7 Harvard Pilgrim Health Care, Boston, MA 02215, USA; denise_simon@harvardpilgrim.org (D.S.); Lauren_Cleveland@harvardpilgrim.org (L.C.); JBLOCK1@partners.org (J.B.)

**Keywords:** calorie, federal labeling law, health policy, interrupted time-series, social media, Twitter, sentiment analysis

## Abstract

Public awareness of calories in food sold in retail establishments is a primary objective of the menu labeling law. This study explores the extent to which we can use social media and internet search queries to understand whether the federal calorie labeling law increased awareness of calories. To evaluate the association of the federal menu labeling law with tweeting about calories we retrieved tweets that contained the term “calorie(s)” from the CompEpi Geo Twitter Database from 1 January through 31 December in 2016 and 2018. Within the same time period, we also retrieved time-series data for search queries related to calories via Google Trends (GT). Interrupted time-series analysis was used to test whether the federal menu labeling law was associated with a change in mentions of “calorie(s)” on Twitter and relative search queries to calories on GT. Before the implementation of the federal calorie labeling law on 7 May 2018, there was a significant decrease in the baseline trend of 4.37 × 10^−8^ (SE = 1.25 × 10^−8^, *p* < 0.001) mean daily ratio of calorie(s) tweets. A significant increase in post-implementation slope of 3.19 × 10^−8^ (SE = 1.34 × 10^−8^ , *p* < 0.018) mean daily ratio of calorie(s) tweets was seen compared to the pre-implementation slope. An interrupted time-series (ITS) analysis showed a small, statistically significant upward trend of 0.0043 (SE = 0.036, *p* < 0.001) weekly search queries for calories pre-implementation, with no significant level change post-implementation. There was a decrease in trend of 1.22 (SE = 0.27, *p* < 0.001) in search queries for calories post-implementation. The federal calorie labeling law was associated with a 173% relative increase in the trend of mean daily ratio of tweets and a -28381% relative change in trend for search queries for calories. Twitter results demonstrate an increase in awareness of calories because of the addition of menu labels. Google Trends results imply that fewer people are searching for the calorie content of their meal, which may no longer be needed since calorie information is provided at point of purchase. Given our findings, discussions online about calories may provide a signal of an increased awareness in the implementation of calorie labels.

## 1. Introduction

As part of the 2010 Affordable Care Act (ACA), Congress passed a national law requiring chain restaurants and similar retail food establishments with 20 or more locations to post calories on menus and menu boards (“menu labeling”). The U.S. Food and Drug Administration (FDA) released final regulatory guidance for menu labeling in 2014, and after several delays, the law went into effect on 7 May 2018. On this date, all large chain retail food establishments (with 20+ sites nationally) were expected to post calories on menus [1]. The federal menu labeling law is an educational intervention for the public and provides important information about the energy content of food and beverages at point-of-purchase. Research evaluating the effects of calorie labeling has examined restaurant transaction databases or collected primary data from individuals in restaurants, cafeterias, and lab settings, with mixed results [2,3,4]. While these studies can assess changes in caloric content of meals along with changes in general perceptions of diners after menu labeling, these studies have not been able to examine larger-scale changes in public perceptions of menu labeling.

Social media provides one method of capturing information about perceptions and norms. Platforms like Twitter, Reddit, and Instagram allow users to document the minutiae of daily life, including dietary intake, and provide a rich source of information about food-related behaviors and perceptions of the U.S. population [5,6] and internationally. In China, recipe search logs from online recipe repositories have been used to model regional cuisine evolution. In one study, the distribution of the types of ingredients used in recipes reflected the migration patterns and changing cultures in that region as well as the influence of changing climate patterns on ingredient use [7]. In another study, using text machine learning algorithms, Amazon food product reviews were associated with U.S. Food and Drug Administration food recalls from 2012 to 2014.[8] Twitter has also provided insight into dietary choices in the US [5]. In one study, mention of eating patterns was extracted from tweets, geolocated using geographic information available on Twitter, and linked to the calorie content of mentioned foods. Higher calorie contents of these foods were correlated with higher regional prevalence of obesity and diabetes [5]. Additionally, when the caloric density of foods mentioned in tweets was examined by U.S. census tract, patterns were similar to dietary trends. Women, people with higher educational attainment, and people in urban areas tweeted about less calorically dense foods. 

Public awareness of calories in food sold in retail establishments is the primary goal of the menu labeling law. Developing a method to understand public perceptions about the federal calorie labeling law can help policy makers understand whether the law is having its intended effects and how, if necessary, to make effective improvements in the future. In the following sections, we explore the extent to which social media and internet search queries reflected the change in menu labeling associated with the federal calorie labeling law and whether the changes are in accordance with the expected increase in awareness of the caloric content of foods.

The objective of this study is to understand the extent to which social media and internet search queries reflected the change in menu labeling associated with the federal calorie labeling law in the United States. 

## 2. Materials and Methods

The main outcome for this study was the ratio of daily tweets containing the term “calorie(s)” to total daily tweets ($\frac{\;\mathrm{Calorie}\;\mathrm{tweet}\;\mathrm{counts}\;}{\mathrm{Total}\;\mathrm{tweet}\;\mathrm{counts}\;}$). This ratio is commonly used in analyses of online big data, such as internet search queries and social media data, to account for changes in the total volume of mentions over time [9,10]. Using Google Trends, we also examined the relative number of queries that included the term “calorie(s)” ($\frac{\;\mathrm{Calorie}\;\mathrm{search}\;\mathrm{queries}\;}{\mathrm{Total}\;\mathrm{Search}\;\mathrm{queries}\;})$ as a secondary outcome. An interrupted time-series design was used to evaluate the potential impact of the federal menu labeling law on tweets about calories and the relatives search volumes for calories on Google Trends.

Twitter data was used to obtain a sample of the social media conversation surrounding calories before and after the implementation of the federal law. Data were taken from the Computational Epidemiology Lab at Harvard Medical School’s Geolocation Twitter Database (CompEpi Geo Twitter Database). Tweets have been collected since 2012 using Twitter’s free Application Programming Interface (Twitter, San Francisco, CA, USA)and a geolocation inference engine developed by the lab [10]. Tweets available include only those for which the user has turned on their geographic positioning system (GPS) location to identify the location where the tweet was created (“geo-tweets”). Opting into the location service enables Twitter to detect the user’s precise location (latitude and longitude) [11]. Overall, approximately 1–3% of tweets from Twitter are available as geo-tweets, amounting to approximately 5 million geo-tweets in the U.S. per day [11]. Although users who opt into pining the GPS location of tweets may not be representative of the U.S. population, previous studies have documented a strong correlation between the number of Twitter users with geo-tweets per state and size of the state population. To obtain more tweets with location information, the CompEpi Geo Twitter Database includes textual information from the users’ profile to collect information about the location. This method has shown high agreement with GPS data [12].

Data was collected from e-retrieved tweets that contained the term “calorie(s)” from the CompEpi Geo Twitter Database from 1 January through 31 December in 2016 and 2018. Data from 2018 were considered the intervention year and those from 2016 were considered the control. Data from 2017 were not included in the analysis because the original menu labeling compliance date was scheduled for May 2017 but was pushed to 2018 on the day before the implementation deadline [13]. At this point, many food retailers had already implemented menu labeling [13,14]. Thus, there may have been an early effect of menu labeling on Twitter discussions of calories in 2017, when select restaurants started labeling. This analysis represents the combined effect of the announcement and national rollout of the federal menu labeling requirements in 2018 [15]. Data from 2017 were less comprehensive than data available in 2016 and 2018. The Institutional Review Board at Boston Children’s Hospital deemed this data exempt.

To assess public awareness of calories before and after the law in another way, we retrieved time-series data for search queries related to calories via Google Trends (GT). Google Trends is a tool that provides information about how frequently a given search term is entered into Google’s search engine relative to the total search volume in that given period. Each data point is divided by the total searches of the time range it represented compared to the relative popularity. The output is then scaled to a range of 0 to 100 based on the topic’s (in this case calories) proportion to all searches on all topics. For the purposes of our study, we restricted the relative search queries to those that were in the U.S. and conducted searches for the term “calorie(s)” in 2016 and 2018.

Slow processing can result in unexpected missing records, from the publicly available Twitter API [16]. To adjust for these interrupted breaks in the data stream, a linear weighted moving average method was applied. This is a common epidemiologic method for filling gaps in Twitter data trends [17]. To calculate weekly rates, each value was aggregated over a 7-day period and was smoothed using a 7-point moving average [17]. We assigned *n* = 7 as the weight for the most recent value, and each prior value as progressively less in linear weight. We multiplied the values for each period by their respective weights to capture the sum total and divided the total by the sum of all the weights to calculate the moving average for each day. This linear moving average imputation method captures the trends in changing values over time. An interrupted value on a given day is x_l_, where l is the index for the date and x is the value for that day. We approximate the interrupted data at x_l_ to be:

(1) $$\frac{\;n\dot{x_{i-1}+\left(n-1\right)x_{i-2}+\left(n-2\right)x_{i-3}+\left(n-3\right)x_{i-4}+\left(n-4\right)x_{i-5}+\left(n-5\right)x_{i-6}+\left(n-6\right)x_{i-7\;}}}{n+\left(n-1\right)+\left(n-2\right)+\left(n-3\right)+\left(n-4\right)+\left(n-5\right)+\left(n-6\right)}$$

To test if the federal menu labeling law was associated with a change in mentions of “calorie(s)” on Twitter, an interrupted time-series analysis (ITSA) was conducted with a single intervention point. The interrupted time-series design is a strong quasi-experimental approach to evaluating the longitudinal effects of population-level interventions [18,19]. We used segmented regression analysis to assess the extent to which the volume of tweets about calorie(s) changed as a result of the federal calorie labeling law. The change point selected in this ITSA, is 7 May 2018, the date that the federal calorie labeling law was rolled out across the U.S. The segments of the time-series are the periods before and after 7 May 2018.

We sought to measure (1) the level of the daily ratio of tweets about calorie(s) and (2) the trend (the rate of change or slope) in the daily ratio of tweets about calorie(s) before and after 7 May 2018.

(2) $$Ratio\;Calorie\left(s\right)\;Tweets_{t}=\beta_{0}+\beta_{1}*day_{t}+\beta_{2}\;\;*\;\;Calorie\;Labeling\;Law_{t\;}+\;\beta_{3}\;\;*\;Post\;Calorie\;Labeling\;Law_{t}+e_{t}$$

where Ratio Calorie(s) Tweets_t_ is the outcome variable, the mean ratio of calorie-related tweets per day to total tweets per day; t is time measured in days at time t from the start of the year; day is the count of days from the beginning the year; Calorie Labeling Law is an indicator for time t occurring before the implementation of the federal menu labeling law (calorie labeling law = 0) or after (calorie labeling law = 1); the law was implemented on the 127th day in the series for 2018; Post Calorie Labeling Law is the number of days after implementation of the law on 7 May 2018. In this model, β_0_ estimates the baseline level of the outcome, mean ratio of calorie(s) tweets per day, at time zero; β_1_ estimates the change in the mean ratio of calorie(s) tweets per day that occurs with each day before the intervention (i.e., the baseline trend); β_2_ estimates the level change in the mean ratio of calorie(s) tweets per day immediately after the intervention, that is, from the end of the preceding segment; β_3_ estimates the change in the trend in the mean ratio of calorie(s) tweets per day after the implementation of the menu labeling law, compared with the daily trend before the menu labeling law. The sum of β_1_ and β_3_ is the post-intervention slope.

Using Model 1 to estimate the level and trend changes associated with the intervention, we control for baseline level and trend, a major strength of segmented regression analysis. The error term e_t_ at time t represents the random variability not explained by the model. It consists of a normally distributed random error and an error term at time t that may be correlated with errors at preceding or subsequent time points. Although the ITS accounts for changes in time trends pre-post implementation of the federal calorie labeling law, we also repeated the time-series analysis for 2016 as the control year prior to implementation. For consistency, in both 2016 and 2018, we used 7 May 2016 as the date of modeled intervention. This analysis was replicated using Google Trends data. A sensitivity analysis was conducted to determine whether the results were robust to the removal of outliers in the data (sharp increases in calorie mentions in November due to the Thanksgiving holiday). 

## 3. Results

In 2018, the mean overall daily number of tweets was 3,494,074 (SD = 917,092) and mean daily number of tweets about calories was 87 (29) (Table 1). The mean daily ratio of tweets about calories to total tweets was 2.4 × 10^−5^ (SD = 9.0 × 10^−6^). In 2016, the mean daily number of tweets was 2,519,899 (SD = 1,208,704) and the mean daily number of tweets about calories was 104 (SD = 52). The mean daily ratio of tweets about calories was 4.1 × 10^−5^ (SD = 9.0 × 10^−6^). Google Trends showed that the mean relative search queries for calorie(s) was 85.52 (SD = 13.22) in 2018 and 79.35 (SD = 12.98) in 2016. These values represent search volume of 79.3% and 85.5% for the day, with the maximum search value in those years. 

**Figure 1:**
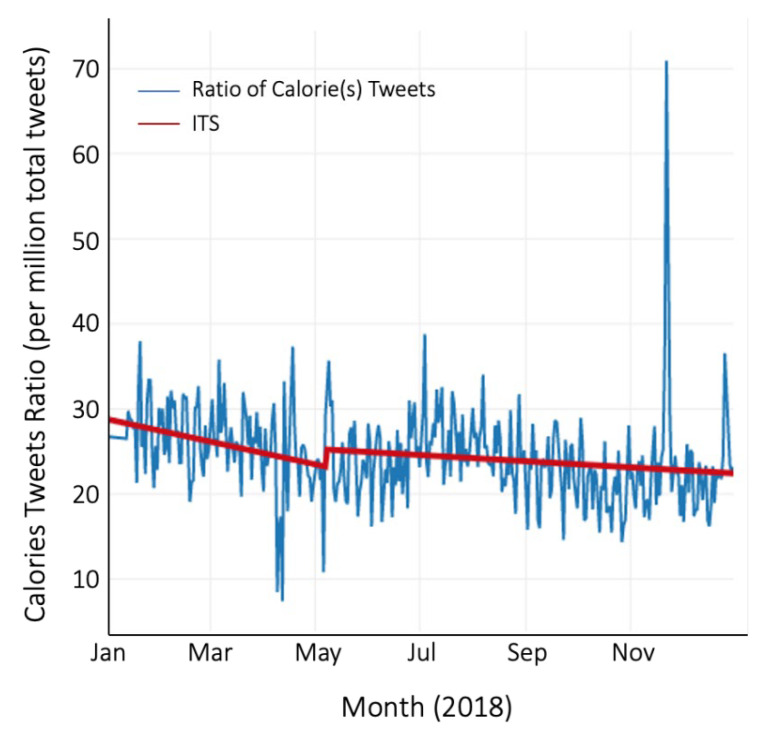
Interrupted Time-series of the daily ratio of calorie(s) tweets in 2018.

**Figure 2:**
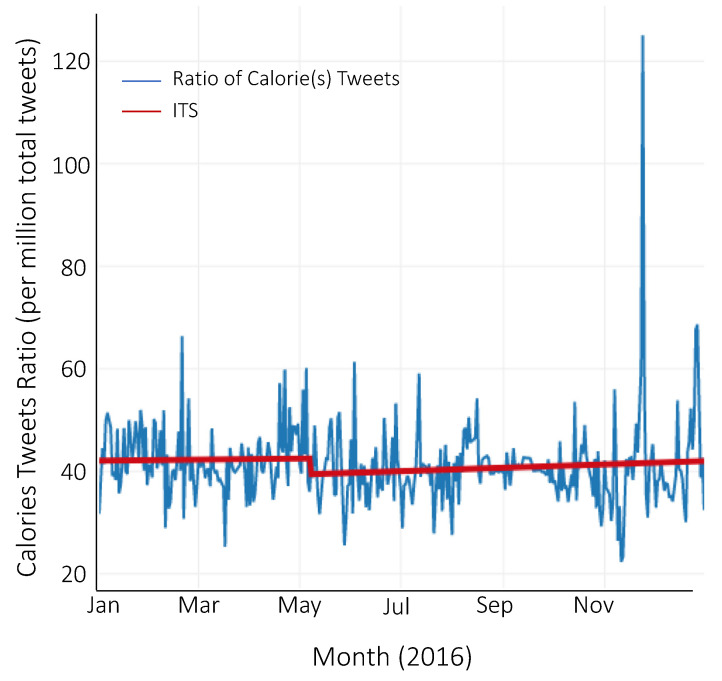
Interrupted time-series of the daily ratio of calorie(s) tweets in 2016.

Before the implementation of the federal calorie labeling law on 7 May 2018, there was a significant decreasing baseline trend of 4.37 × 10^−8^ (SE = 1.25 × 10^−8^, *p* < 0.001) mean daily ratio of calorie(s) tweets (Table 2, Figure 1). There was no level change post-implementation. There was, however, an increasing post-implementation slope of 3.19 × 10^−8^ (SE = 1.34 × 10^−8^, *p* < 0.018) mean daily ratio of calorie(s) tweets compared to the pre-implementation slope; equivalent to a 173% increase in trend. This post-implementation slope is still negative; however, it represents an increase in trend (or less negative) compared to the pre-implementation slope. When using the corresponding date of 7 May as the inflection point for 2016, we see a decreasing baseline trend in the mean daily ratio of calorie(s) tweets before 7 May 2016 (Table 2, Figure 2). However, unlike in 2018, we found no change in either the level or trend post 7 May; the trend in the pre- and post-implementation periods is similar. All results were robust to sensitivity analyses when outliers were removed.

**Figure 3:**
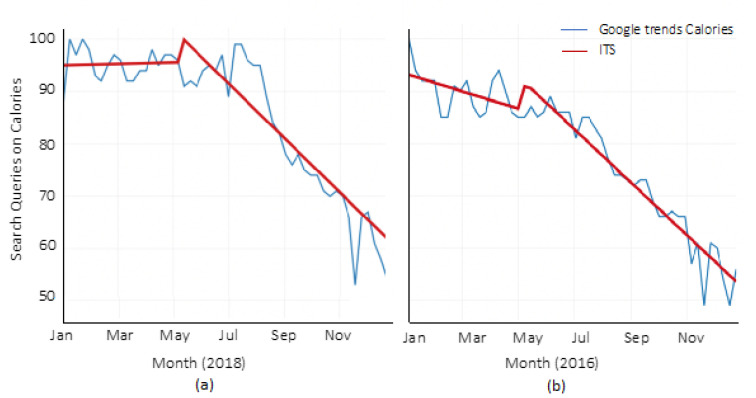
Interrupted time-series for Google Trends on Calories. (**a**) Year 2018, (**b**) Year 2016.

In 2018, Google Trends search queries for calories appeared fairly stable pre-implementation but declined substantially post-implementation (Figure 3). Patterns were similar in 2016, but search queries for calories were lower this year compared to 2018 and did not decline in the same pattern post-implementation. The ITS analysis showed a small, statistically significant upward trend of 0.0043 (SE = 0.036, *p* < 0.001) weekly search queries for calories pre-implementation, with no significant level change post-implementation (Table 3). There was a decrease in trend of 1.22 (SE = 0.27, *p* < 0.001) search queries for calories post-implementation. In 2016, there was a decreasing trend of 0.055 (SE = 0.023, *p* < 0.001) weekly search queries for calories before 7 May, with a significant level change of 4.64 (SE = 2.17, *p* < 0.001) after 7 May. A significant decrease in trend (0.77, SE = 0.17, *p* < 0.001) was seen after 7 May and was less steep compared to 2018. 

## 4. Discussion

This analysis of mentions of calories on Twitter showed that the implementation of the federal calorie labeling law on 7 May 2020 was associated with a 173% relative increase in the trend of mean daily ratio of tweets. Overall, this led to a post-implementation trend of mean daily ratio of tweets that were less negative than pre-implementation. In the comparison year of 2016, we observed no significant level or trend changes after 7 May 2016. 

Our Google Trends analysis revealed substantial trend changes in relative search queries for calories post-implementation in 2018. In 2016, after 7 May, a significant increase in level change was seen followed by a significant decrease in trend for relative search queries. These results potentially suggest that fewer people are searching for the calorie content of their meal, which may no longer be needed since calorie information is provided at point of purchase. This is consistent with studies showing that people are better able to accurately estimate calories in their meal when calories are posted on the menu [20,21,22]. Another possible reason for the decline in queries after menu labeling implementation could be declines in news coverage or searches by restaurants for implementation guidance. 

Our results showing increasing trends in calorie mentions on Twitter following calorie labeling are consistent with prior research showing that calorie labeling affects some of the food choices among those who are aware of calorie labels [23]. Additionally, evidence has demonstrated that front-of-package labels can help persons assess nutritional information better than when no-label is present [20]. Given our findings, discussions online about calories may provide a signal of increased awareness to the implementation of the calorie labels.

Although a significant increase in trend was seen post-implementation, we did not observe a level change in mentions of calories on Twitter. One possible explanation for this lack of effect was the gradual rollout of menu labeling in restaurants and similar food retail establishments over time. McDonald’s is the nation’s top revenue-generating restaurant with a brand value of about 156 billion U.S. dollars in 2019 [24] and the largest market share of fast-food restaurants with 43% of the U.S fast-serve market [25]. McDonald’s implemented menu labeling nationwide in 2012. Other large national chains implemented labeling prior to the federal implementation deadline to comply with state or local laws [26]. Some chains began labeling prior to May 2018 in anticipation of the expected earlier deadlines for implementation, most notably in May of 2017 [27]. Labeling may not be enough by itself to promote awareness and discussion of calories. Large-scale mass media campaigns may be needed to promote greater dissemination of this information to the public [28,29,30]. For example, Chile’s Law of Food Labeling and Advertising, implemented in 2016, jointly mandated front-of-package warning labels and a mass media educational campaign to restrict child-directed marketing; the law has been associated with reduced purchasing of high-calorie beverages [31,32]. The U.S. FDA did not release consumer-facing educational campaign on the date of menu labeling implementation, so when educational resources were released in 2019, there was limited reach [15].

Furthermore, as of October 2018, an estimated total daily volume of Twitter has reached the same height as in June 2012 [33]. Twitter remains a hugely relevant culture force, whereby topics related to activist movements such as Me Too, March For Our Lives, and Black Lives Matter, and discussions about political figures such as Donald Trump, Barack Obama, Hillary Clinton, were highly popular in 2018 and may have reduced the relative ratio of tweets about calories compared to 2016 [34]. 

Our study has some limitations. The sample population may not be representative of the U.S. population. However, Twitter has been successfully used to predict stock market trends [35] and political elections,[36] indicating its generalizability and ability to assess population-based trends. Additionally, this study used the keyword “calorie(s)” for Twitter and search queries which may not differentiate the potential intake from outtake and thus may be limited in its face validity for caloric consumption alone. The addition of specific keywords such as calorie “menu” or “label” resulted in a very small number of tweets per week. The small sample space lends itself poorly to broad conclusions regarding the overall twitter sentiment. Qualitative observation of the tweets about calories shows that the majority of the tweets are centered around referencing a specific item rather than the general use of the word “food”, “menu” or “label”. For example, tweets do not mention “food”, but rather a specific food item such as “oreo package: only 140 calories per 4 cookies!”, “I start my 1500 calorie diet today with 2 cups of veggies and 2 cups of greens” and “What could possibly be bad about having Two Big Macs, Two Filet-o-Fish, One Lg fries 4 lunch? 2400 calories.”

There is no single additional keyword that would allow us to narrow the scope of the tweets while still capturing all relevant data. Therefore, we accept this tradeoff as a limitation of the Twitter data. We acknowledge that the results of the study may be shaded by tweets that are not directly relevant to the calorie discussion that we are centering. Descriptive analysis of the tweets show that these irrelevant calorie tweets make up a very small subset of the total data, maintaining face validity of using the broad “calorie” search term in the study. Finally, data collected from Google Trends comparing the terms of “calorie menu”, “calorie food”, and “calorie meal” showed that relative to the search term “calorie” was negligible, indicating that searches for calories are used in a broader context.

The goal of this study was to better understand the broader impact of how the new federal labeling law may have influenced the focus on calories in the context of a greater concentration and focus of calories at the input and output levels. Future analyses should focus on food groups or items to identify the impact of the labeling law on specific meal/food consumption.

Furthermore, a relative scale is used to evaluate social media and search trends because of the lack of ability to interpret absolute data trends. This method is consistent with previous studies that have conducted relative comparisons against controls to better interpret and understand these Twitter based results. For instance, studies that evaluated the effect of the implementation of the Affordable Care Act using the relative change in patient experience sentiment of users on Twitter [37].

## 5. Conclusions

Our study demonstrates the ability of social media and search queries to evaluate the implementation of national nutritional policies. The federal menu labelling law prompted changes that were subsequently reflected in twitter discourse, providing insight into consumer attitudes and awareness. Future studies should further investigate the relationship between consumer behavior and nutritional laws, in order to provide policy makers feedback to improve the effectiveness of these policies through an iterative design in real-time.

## Figures and Tables

**Table 1:** Descriptions of tweets about calories in 2018 (implementation of federal calorie labeling law) and 2016 (comparison year).

2018	Calorie(s) Tweets	Total Daily Tweets	Ratio of Daily Calorie(s) Tweets to Total Tweets
Mean	87	3,494,074	2.4 × 10^−5^
STD	29	917,092	6.0 × 10^−6^
Min	1	110	7.0 × 10^−6^
25% Quantile	73	3,502,201	2.1 × 10^−5^
50% Quantile	85	3,585,176	2.4 × 10^−5^
75% Quantile	97	3,638,652	2.7 × 10^−5^
Max	252	7,172,789	7.1 × 10^−5^
**2016**	**Calorie(s) Tweets**	**Total Daily Tweets**	**Ratio of Daily Calorie(s) Tweets to Total Tweets**
Mean	104	2,519,899	4.1 × 10^−5^
STD	52	1,208,704	9.0 × 10^−6^
Min	2	47,064	2.2 × 10^−5^
25% Quantile	61	1,565,400	3.7 × 10^−5^
50% Quantile	102	2,442,214	4.0 × 10^−5^
75% Quantile	149	3,790,938	4.4 × 10^−5^
Max	219	4,119,771	1.3 × 10^−5^

**Table 2:** Descriptions of relative Google search queries about calories in 2018 (implementation of federal calorie labeling law) and 2016 (comparison year).

	2018	2016
Mean	85.52	79.35
STD	13.22	12.98
Min	54	49
25% Quantile	76	69.25
50% Quantile	92	85.5
75% Quantile	95	88.25
Max	100	100

**Table 3:** Parameter estimates, standard errors and *p*-values from the fitted segmented linear regression models of daily mean calorie(s) tweets in 2018 and 2016.

	Coefficient	Standard Error	T-Statistic	*p*-Value
	2018			
Baseline level β_0_	7.95 × 10^−4^	2.20 × 10^−4^	3.611	<0.001
Baseline trend β_1_	−4.37 × 10^−8^	1.25 × 10^−8^	−3.493	<0.001
Level change post-implementation β_2_	2.01 × 10^−6^	1.13 × 10^−6^	1.772	0.077
Trend change post- implementation β_3_	3.19 × 10^−8^	1.34 × 10^−8^	2.373	0.018
	2016			
Baseline level β_0_	−1.09 × 10^−5^	3.15 × 10^−4^	−0.034	0.973
Baseline trend β_1_	3.15 × 10^−9^	1.87 × 10^−8^	0.169	0.866
Level change post-implementation β_2_	−3.05 × 10^−6^	1.71 × 10^−6^	−1.786	0.075
Trend change post-implementation β_3_	7.48 × 10^−9^	2.01 × 10^−8^	0.372	0.710

β_0_ estimates the baseline level of the outcome, mean ratio of calorie(s) tweets per day, at time zero; β_1_ estimates the change in the mean ratio of calorie(s) tweets per day that occurs with each day before the intervention (i.e., the baseline trend); β_2_ estimates the level change in the mean ratio of calorie(s) tweets per day immediately after the intervention, that is, from the end of the preceding segment; β_3_ estimates the change in the trend in the mean ratio of calorie(s) tweets per day after the implementation of the menu labeling law, compared with the daily trend before the menu labeling law.

## Data Availability

Data was obtained from Twitter and are publicly available from Twitter’s API.
